# Exploring Black British African and Caribbean peoples' experiences of self‐harm and accessing support

**DOI:** 10.1111/papt.70063

**Published:** 2026-05-18

**Authors:** Olivia Alleyne, Vyv Huddy

**Affiliations:** ^1^ Symbol Therapy Team for Children in Care and Adopted Children Lewisham CAMHS London UK; ^2^ School of Psychology University of Sheffield Sheffield UK

**Keywords:** Black British African and Caribbean, interpretative phenomenological analysis, qualitative, self‐harm, third sector

## Abstract

**Introduction:**

It is important to understand the underrepresented experiences of self‐harm and accessing support among Black British African and Caribbean individuals because of the low uptake of support from formal healthcare providers. This study aimed to explore Black British individuals' thoughts, feelings and experiences of self‐harm, and to understand their lived experience of seeking, or not seeking, support from voluntary and community sector organisations.

**Method:**

Purposive sampling was utilised to identify Black British people that self‐harm and have either accessed community‐based services or never sought support. Six people took part in semi‐structured interviews. Interpretative Phenomenological Analysis was used to analyse the data.

**Results:**

Three group experiential themes were found. Firstly, the complexities of self‐harm manifested in diverse ways and was something participants had a complex relationship with. Secondly, participants described navigating expectations; they concealed self‐harm to protect against stigma and to uphold valued cultural norms of strength and success. Finally, promoting equity: community‐based services were a preferred means of support but noted unaddressed hardships and a need for more culturally sensitive support.

**Discussion:**

Findings highlight the need for community‐based services to develop equity‐focused resources to achieve more culturally responsive care. Key findings, limitations and implications are discussed in relation to existing theory and suggestions for future research are made.

## INTRODUCTION

Self‐harm is a widespread public health concern (Mughal et al., 2023) defined by the National Institute for Health and Care Excellence (NICE) as ‘intentional self‐poisoning or injury, irrespective of the apparent purpose’ (NICE, 2022, p. 6). While over 73,000 people received emergency care in England for intentional self‐harm in 2023 alone (Office for Health Improvement and Disparities, 2024), extant literature suggests that many incidents of self‐harm are under‐reported and undetected (Geulayov et al., 2018; McManus et al., 2019).

Self‐harming behaviours according to race^1^ and ethnicity have been infrequently explored. That said, robust research has shown disproportionately elevated rates of self‐harm among Black British African and Caribbean women compared to their counterparts from other ethnic and racial backgrounds (Al‐Sharifi et al., 2015; Cooper et al., 2010). These findings may, in part, be attributed to the lived experiences of racism and marginalisation (Chakraborty et al., 2010), as well as the compounding effects of socioeconomic inequalities, such as poverty (Bamford et al., 2020; Mangalore & Knapp, 2011). Importantly, these disparities in self‐harming behaviours align with the well‐documented high prevalence of mental health challenges, including depression, within Black British populations (Halvorsrud et al., 2019). It is important to note that the persistent, everyday, experience of racism has also been understood as cumulative, much as weather erodes rock, leading to higher rates of physical and mental health conditions (Geronimus, 1992).

Yet, despite the disproportionately higher rates of self‐harm and mental health challenges, Black British individuals remain underrepresented in primary care services and talking therapies (Morris et al., 2020). Hence, questions arise in relation to Black British individual experiences of help‐seeking. Some deterrents to accessing support have been identified in the literature and include fears of encountering racism and a perceived lack of cultural understanding within mainstream service providers (Keating & Robertson, 2002). In response to deterrents to accessing formal health support, many Black British individuals have expressed a preference for community‐based support networks (Sisley et al., 2011), more formally known as the third sector.^2^

Community‐based organisations show promise in acting as a more trusted and culturally appropriate support alternative (Baskin et al., 2021). Consequently, attention is shifting to the role of community‐based support and its ability to provide effective care for self‐harm (Hulin et al., 2024). Studies indicate that community‐based organisations have successfully addressed social isolation (Calò et al., 2021) and improved depression (Afuwape et al., 2010) for people of the Global Majority.^3^ However, there are still knowledge gaps in understanding barriers to accessing community‐based organisations or what resources are needed to support Black British individuals that self‐harm.

A theoretical context relevant to why some Black British individuals may choose to not access mental health support is the concept of ‘discourse of strength’ (Beauboeuf‐Lafontant, 2008). This idea sheds light on how culturally reinforced expectations of self‐efficacy and resilience may discourage vulnerability. Such discourses are suggested to be rooted in the historical oppression of slavery, whereby Black African and Caribbean people were forced to develop survival mechanisms to withstand hardships beyond what is considered ‘normal’ (Watson‐Singleton, 2017). Over generations, cultural expectations of strength in the face of adversity may have become internalised in concepts prevalent today, such as the ‘strong black woman’ (Parks & Hayman, 2024). Lending empirical support, Edge's (2007) study revealed how psychological wellbeing was maintained by Black Caribbean women navigating perinatal depression through a strong internal locus of control.

Self‐harm appears to hold intricate and personal meanings that vary for those who engage in it (Lloyd‐Richardson et al., 2007; Scoliers et al., 2008; Simopoulou & Chandler, 2020). It is no surprise, then, that exploration into the subjective experience of self‐harm has uncovered complex psychological functions (Edmondson, 2013; Sutton, 2007). One commonly reported function of self‐harm is affect regulation (Kuehn et al., 2022; McKenzie & Gross, 2014). To elaborate, this is where self‐harm serves to manage negative emotional and cognitive intra‐personal experiences (O'Shea et al., 2019). From this perspective, self‐harm can act as a means to cope with emotions, such as sadness or anger, that feel intolerably overwhelming for the individual (Tofthagen et al., 2021).

Psychoanalytic theories also offer insightful perspectives on the communicative functions of self‐harm. One view, as presented by Favazza (1996), is that scars can express distress that are challenging to articulate. This theoretical perspective closely relates to the supposition that self‐inflicted pain can validate, and demonstrate, private psychological suffering through making the invisible, visible (Warm et al., 2003). Additionally, Edmondson et al. (2016) systematic review revealed that participants' experiences of self‐harm were positive and pleasurable, further demonstrating its complexity. The research focused on the meaning or function of self‐harm has not made explicit reference to Black African and Caribbean communities, which are underrepresented in research (Smart & Harrison, 2017), and this is likely to weaken its relevance to understanding difficulties experienced by individuals from this community.

Research from the Global South highlights how cultural, economic, and structural contexts shape understandings of self‐harm and pathways to support in ways that are not always captured in Global North research. A recent systematic review by Jidong et al. (2024), examining interventions for self‐harm and suicidal ideation across African contexts. The review highlights how stigma, culturally specific meanings, and barriers to accessing formal support influence help‐seeking. These insights are relevant for understanding the experiences of Black British individuals, particularly those from first and second generation backgrounds, whose constructions of self‐harm and help‐seeking may be shaped by intergenerational cultural frameworks and migration histories, alongside their navigation of UK‐based support systems.

Research exploring self‐harm within Global Majority communities primarily captures experiences within child and adolescent populations (Burgess et al., 2022; Farooq et al., 2021; Rehman et al., 2020). Furthermore, access is traditionally examined from the point of entrance to formal services (Memon et al., 2016; Rabiee & Smith, 2014). However, it may be better conceptualised as a process beginning long before the point of entry (Levesque et al., 2013). There is extremely limited literature capturing the voices of adults who self‐harm and have never sought formal or informal support. The present study, therefore, recognises a need to understand these experiences to gain a more holistic understanding of the barriers and facilitators to help‐seeking.

### Research aims

Evidently, greater insight is required into how Black British individuals who engage in self‐harm access and utilise community‐based support services. While extant literature has provided quantified rates of the prevalence of self‐harm in Black British communities (Cooper et al., 2010), qualitative research is needed to explore the lived experience in these communities. This is crucial for developing responsive, equitable and culturally competent services that can appropriately address mental health needs and self‐harming behaviours.

The study aims to address the gaps in the literature by exploring Black British individuals' thoughts, feelings and experiences of self‐harm, and to understand their lived experience of seeking, or not seeking, community‐based support. To the best of the author's knowledge, this is the first study that aims to capture the voices of Black British individuals and their experiences of self‐harm.

### Research questions

The aims of this study have resulted in three main research questions:

- How is self‐harm experienced by individuals within the Black British ethnic group?
- How are ideas of self‐harm constructed and viewed by individuals within the Black British ethnic group?
- What are the barriers and facilitators for accessing community‐based support for self‐harm within the Black British communities?

## METHOD

### Epistemological framework

The author sits within the perspective that conditions and attitudes exist independent of an individual's understanding, while maintaining that knowledge and interpretation of these conditions are subjective and negotiated through social interaction (Gray, 2018). Compatible with this view is the epistemological position of critical realism (Bentall, 1999). Within the assumption that knowledge is local and context‐dependent, critical realism reflects a phenomenological approach seeking to understand the lived experiences (Madill et al., 2000), and has the capacity to translate into person‐centred approaches within clinical psychology (Hauser, 2023). It is therefore the chosen framework for this study.

### Design

This study is grounded in the perspective that individuals ascribe unique and personal meanings to their lived experiences. One methodology concerned with generating an in‐depth understanding of a person's lived experience, as well as the meaning they attach to it, is Interpretative Phenomenological Analysis (IPA). The present study lies within IPA's strong emphasis on individual experience. This allows for a deep exploration into how discourses influence, and are influenced by, intra‐personal and inter‐group dynamics. Its capacity to empower marginalised voices (Orbe, 2000) places IPA as an appropriate option for answering the research questions.

### Reflexivity

For transparency, the researcher is a mixed Black Caribbean and White woman raised within British norms. She has an intimate familiarity with these topics of navigating marginalisation and lack of familiarity in accessing mental health services for mental health challenges or self‐harm. The researcher is also a Clinical Psychologist. Olmos‐Vega et al. (2023) states the importance of going beyond describing one's background. A reflexivity journal was used to heighten the researcher's awareness of sense of self and to navigate the experience of being an insider culturally, but an outsider professionally. The second author is a White male experience academic raised in a northern English city. He fostered an interest in the barriers preventing Black African and Caribbean people from equitable access to mental health care whilst a clinical psychologist in inpatient mental health services and the criminal justice system.

### Patient and public involvement (PPI)

The PPI contributor identified as a Black mixed African and Caribbean female who experienced self‐harm. Together, all resources were reviewed at the design stage and a pilot interview was completed. From this, invaluable constructive feedback was received on the interview schedule, information sheet and consent form. For example, it was highlighted that wording on the interview schedule could be changed from ‘black community’ to ‘Afro‐Caribbean community’. Amendments were then made.

### Ethics

Ethical approval was obtained from the University of Sheffield Research Ethics Committee (Reference Number 053155) and abided by the University's Research Ethics Policy (The University of Sheffield, 2019).

### Recruitment and participants

IPA advocates using a reasonably homogeneous and smaller sample to allow for highly detailed analysis (Smith et al., 2009). Homogeneity, in this context, does not imply that all participants have identical experiences; rather, it refers to the shared experiences related to the research topic (Smith et al., 2009). The study employed a purposive sampling strategy based on the theoretical grounds that Black British people are more likely to utilise community‐based services than statutory services (Keating & Robertson, 2004; Mclean et al., 2003). Specifically for this study, Black British individuals with relevant experiences and perspectives of self‐harm and community‐based service use were recruited according to the inclusion criteria (Table 1).

**TABLE 1:** Inclusion and exclusion criteria.

Inclusion criteria	Exclusion criteria
Adults aged 18+	Individuals who identified as being from different ethnic backgrounds (e.g., Asian, Pakistani, Chinese, White, European)
Individuals who identify as Black British African and/or Caribbean or Mixed Black African and/or Caribbean	Individuals whose behaviour is not regarded as deliberate self‐harm, for example, repetitive head banging with individuals with learning disabilities
Self‐Identifies as having lived experience of self‐harm defined by NICE (2011). For example, any act of self‐poisoning or self‐injury… irrespective of motivation. This was stated on the advert.	Individuals unable to provide informed consent
Has accessed third‐sector organisations or has never accessed any mental health support	Individuals who were unable to read or speak English

The researcher sent E‐mails to third sector organisations across England. These services included self‐harm charities, Global Majority community groups and mental health social enterprises. Organisations were asked to circulate the study advert to potential participants through their existing communication channels, such as forwarding via e‐mailing lists or internal email networks. The advert was also shared on social media platforms, such as Linkedin and Facebook groups for people who self‐harm.

Over the course of 4 months, 11 people responded to the advert. Eligibility was assessed via email correspondence where respondents confirmed they met the inclusion and exclusion criteria outlined in the study advert. One individual met the exclusion criteria of being under 18 years old, and four did not respond at the stage of interview arrangement. Overall, six participants considered appropriate for the research aims took part in the study; a number recommended for doctoral IPA studies (Smith et al., 2009). No prior relationship existed between the researcher and participants before initial contact; however, the study advert included the researcher's photograph, academic role, and a brief description of the study aims. A summary of participant demographic and contextual information is presented in Table 2.

**TABLE 2:** Participant demographic information.

Participant	Gender	Birthplace	Years lived in UK	Ethnicity	Support accessed	Self‐harm category (NICE, 2011)
1	Gender non‐conforming	England	Since birth	Black African	Third sector	Cutting self‐poisoning suicide attempts starving
2	Male	Africa	>15	Black African	Third sector	Self‐poisoning suicide attempt
3	Female	England	Since birth	Mixed Black African or/and Caribbean	None	Cutting
4	Female	England	Since birth	Black Caribbean	Third sector	Cutting
5	Female	England	Since birth	Mixed Black African or/and Caribbean	Third sector	Cutting
6	Male	Europe	>15	Black African	None	Self‐poisoning excessive exercise vomiting

Informed consent was obtained electronically following eligibility confirmation and prior to data collection. Participants were sent a participant information sheet via email and asked to complete an online consent form hosted on Qualtrics. The email invited participants to ask questions before agreeing to take part and to indicate their availability for an interview. Consent was verbally reconfirmed at the beginning of each interview.

### Data collection

Semi structured interviews were conducted remotely using the secure video conferencing platform Google Meet. The interview schedule was informed by guidance (McIntosh & Morse, 2015), and existing topics in the literature. It explored participants' lived experiences and perceptions of self‐harm, including emotional responses and experiences of seeking and engaging with support services. The interviews were anonymised and transcribed verbatim by a single transcriber within the University's transcription service. The interviews lasted between 39 and 64 min (mean = 51.5 min). Following the interview, a verbal debrief was offered and the debrief form was sent. Participants fed back that the study felt ‘respectful’ and as though the researcher was ‘listening, not just interviewing’.

### Analysis

Data analysis followed Smith et al. (2022) guidance for conducting IPA. The researcher conducted the interviews and then listened to each interview, first without the transcript and again with it, to ensure familiarity with not just what the participant said, but how they said it. The transcript was inputted into the qualitative data analysis computer software NVivo 14 for Mac. NVivo was employed to facilitate the organisation and maintenance of the data. Here, the researcher made line‐by‐line exploratory comments focusing on descriptive, linguistic, and conceptual aspects of the participant's experience.

One criticism of IPA studies is how they can get ‘stuck’ at the first‐order level of analysis in only summarising participant sense making (Larkin et al., 2006). Therefore, to support the development of the researcher's interpretations, the researcher used the ‘annotation’ feature in NVivo to anchor reflections in the transcripts and transition into the researcher's sense‐making.

The comments from each transcript were merged according to patterns of meaning to construct experiential statements which were grouped into personal experiential themes. This process was repeated for the remaining five interviews. Then, the experiential themes were printed on to coloured paper, with each colour representing a different participant. The themes were then organised visually within cautious cross‐case analysis. From this, group experiential themes were refined. The trustworthiness of rigour of the findings was guided by the criteria set out by Elliott et al. (1999). The second author co‐analysed one transcript and engaged in extension discussion with the first author about final themes and interpretations.

## RESULTS

A total of three group experiential themes emerged from the data analysis (Table 3). An interpretive account of each theme is introduced and summarised. Individual voices are presented through verbatim quotes. Importantly, these themes, while chosen due to participant value and significance, do not encapsulate the full diversity of Black British experiences of self‐harm and help‐seeking. Aspects of experience overlap themes. See Table 3 for participants' contributions according to each theme.

**TABLE 3:** Themes and contribution to themes by participant.

Group experiential theme	Subthemes	P1	P2	P3	P4	P5	P6
Complexities of self‐harm	More than a stereotype	✔	✔	✔	✔	✔	✔
	Refuge from overwhelm	✔	✔	✔		✔	✔
	‘It is a love–hate relationship’	✔	✔	✔	✔	✔	✔
Navigating expectations	Feeling othered	✔		✔	✔	✔	
	Concealing for self‐protection	✔	✔	✔	✔	✔	✔
	Keeping it moving	✔	✔	✔		✔	✔
Promoting equity	Unaddressed hardships	✔		✔	✔	✔	✔
	Person centred care—‘We're not a Monolith’	✔	✔	✔	✔	✔	✔
	Helpful systems	✔	✔		✔	✔	✔

The researcher considered the relationship between the themes and presents them in a way that mirrors some of the participants' journey to support.

### Complexities of self‐harm

Within this theme, the multifaceted nature and presentation of self‐harm is captured. It explores diverse manifestations and motivations as shared by participants. Participants had a complex relationship with self‐harm, as it was not experienced as a single or static behaviour; rather, something that took multiple forms, shifted over time and provided both relief and further distress.

#### More than a stereotype

All participants spoke to their use of a broad range of behaviours that knowingly caused emotional or physical damage to themselves. It was highlighted that self‐harm is ‘not always the stereotypical ways that we think of’ (P6) and can manifest through acts that are socially unrecognised and unacknowledged by services:

I think people think of self‐harm as the stereotypical maybe cutting with, you know, a razor but actually, you know, it can look like starving or using different instruments or, things that are really detrimental to your health, so all those things can be self‐harm, but I don't think they're always seen as that. (P3)

For most participants, these diverse forms of self‐harm were co‐occurring and included intentional cutting, starvation, excessive exercise, vomiting and self‐poisoning. While cutting was described as more visible and socially recognised, several participants described forms of self‐harm that were enacted through withholding care from the body:

…I just wouldn't eat … I wasn't taking care of myself, and it would bring me physical pain being in the state where I wasn't giving my body nutrients and I would just be in intense pain because I would get these stomach cramps. (P1)

These practises were often less immediately recognisable as self‐harm and, in some cases, only became identifiable once physical or psychological effects accumulated over time. One participant described engaging in future oriented forms of self‐harm that was enacted through escalation rather than immediate physical harm:

…trying to take more alcohol and drugs so that hopefully I will become an addict and try to hurt myself the most by commit suicide. (P2)

#### Refuge from overwhelm

Conveyed across all participants was that self‐ham was self‐soothing when faced with life stressors, overwhelm and depression. It was perceived as a ‘side effect of the really fast paced society that we live in’ (P3):

I felt like I had to do it because it gave me some kind of, it was like a sense of release from every, all the emotions and the thoughts that I was keeping in, so it actually became that release but something that I felt that I had to do. (P3)

For most, the act provided participants with a sense of release, and was considered satisfying though its ability to numb and distract participants from emotional distress:

I remember just feeling this like deep sense of dread and doing it kind of just distracted the thoughts, it made me focus in on something that took me away from that … I think it just gave me a break away from that feeling honestly. (P1)

A sense of accomplishment was experienced by participants when self‐harm was used to respond to triggers of emotional distress. This left some participants feeling empowered when self‐harming.

…there being a feeling of satisfaction because it felt like I was actually doing something about these feelings… it came with that sense of accomplishment cause I could see the result after it. (P4)

#### It is a love–hate relationship

Self‐harm was something participants had ‘a love–hate relationship with’ (P4) and this relationship was paradoxical and ever‐changing in nature. While self‐harm was employed to manage distress, perspectives revealed how satisfaction was temporary and thwarted by its inability to fully resolve inner conflict, deeming it a ‘short‐term solution to a long‐term problem’ (P3):

If you're not addressing the issue and just putting a plaster on whatever the issue, a broken leg right, what's the plaster gonna do? (P6)

Within these insights, participants identified unhelpful consequences of self‐harm that invited further frustration and barriers, such as regret and having to hide parts of their body from friends and loved ones. Thus, further complicating the relationship they had to themselves and experiencing it as ‘a vicious cycle’ (P1)

like it was fine at the start and then after I would be like really upset about it and then it would regret it. (P5)

In this way, self‐harm was viewed by participants as an unhealthy way to manage distress, claiming ‘It's in the name, its harm, it's not doing any good’ (P4). While this was recognised, the experience of self‐harm, however, was described as addictive:

…It was the best way for me to get addicted to this. I try to get emotional things away or try to, to stop overthinking regarding to this incident, so I tried to involve myself into all this and tried to hurt myself more and more. (P2)

One participant described how her relationship with self‐harm intensified. Here, self‐harm satisfaction was only achieved when the act matched the expectation of pain:

I wasn't able to cut deep enough. With it being more painful it felt like what I was doing was actually working and when it wasn't it annoyed me cos I had this expectation that it would bleed a lot more and when it wouldn't, I would start like putting salt into my wounds just so I could see them more. (P1)

### Navigating expectations

This theme explores participants' constructions of self‐harm in the context of sociocultural norms and expectations. Participants noticed self‐harm occurring in response to marginalisation and described a process of concealing their struggles to manage the opinions of others and uphold traditions that prioritised strength and resilience.

#### Feeling othered

From a young age, experiences of racism and marginalisation played an integral role in participants' identity formation. This emerged as a trigger for self‐harm for four participants. Their visible racial differences felt socially ‘othered’ within predominantly White environments. A lack of perceived belonging fostered internalisations of themselves as ‘different’ (P4), which was experienced as socially isolating and generated fears of social exclusion.

Being different I'd always assume would make people not want to be around me and that would mean I would be alone. (P1)

For some participants, these experiences of racialised bullying and stereotypes were directly linked to self‐harm, as repeated exposure to racism and social rejection contributed to feelings of worthlessness and distress:

I am who I am because of the sh*t I took, you know, the bullying the jokes about liking chicken, all of it. (P3)

Feeling ‘othered’ was compounded by societal preferences for White beauty standards and further exacerbated marginalisation for some. Three participants described a pressure to conform to these standards, which negatively impacted self‐perceptions:

I was the only person in my year who was Black so I wanted to kind of fit in and simulate being a certain size, shape, hair type, you know, that was something when I was growing up that was really quite influential. (P3)

Participants recalled how they began to punish themselves in response to their lack of proximity to Whiteness. Here, physical attributes, such as skin colour and hair type, were regarded as flaws deserving of harm. One participant resorted to starving themselves ‘to correct my Black qualities’ (P.5). For another, perceived worthlessness acted as a founding reason for cutting themselves”

I wasn't White British so I didn't have straight hair, you know, for me the self‐harm was almost erm, almost like a punishment for not being those things. (P4)

#### Concealing for self‐protection

Within the family context, Black British community, and wider society, participants unanimously described a lack of accurate understanding of mental challenges or self‐harm. Here, participants detailed a difficulty in navigating the views and attitudes of others:

There is still a lot of stigma which does prevent people from reaching out, whether its to friends or to family or professional support or, you know, hiding their scars, making excuses to how they maybe got the particular cut. (P3)

A deep reluctance to discuss their experiences with others was observed within participants' narratives. One of the reasons participants felt they had to ‘protect against other people's opinions’ (P.5) was because society's view that mental health means you are ‘crazy’ (P6).

Participants also spoke of generational differences in attitudes toward mental health difficulties, finding that older family members tended to have more stigmatising views. The stigmatic environment created an inability for participants to be wholly transparent about their struggles to their parents:

I think like mental health like the awareness is very new and recent and having like an old‐fashioned African mum, there is certain things that you don't really speak about to them and mental health and self‐harm is one on the top list. (P5)

In examples where participants did try to disclose aspects of their distress to their wider support systems, some responses were considered unhelpful, leaving them feeling even more isolated, ‘shut down’ (P6) or ‘judged’ (P3):

When I was in pain, erm I think a lot of thought comes in and people try to neglect me. I try to come close to certain individuals to try to explain to them but because they think I'm a failed individual they try to neglect me, they try to neglect my attention, they try to, to avoid me. (P2)

Participants went to great lengths to conceal their self‐harm reality. One participant transitioned across different methods of self‐harm to produce an illusion of control that doubled as a strategy to reduce the likeliness of acts being discovered by others.

I make myself puke, you know, I do it to hurt myself, I realised that this was just not healthy for me, but then I transitioned to something else. I just replaced one way with another so I could tell myself, you know, “well I stopped doing that type”. It's a delusion, as well probably to, to make, sure no one could catch on. (P6)

#### Keeping it moving

Traditions, norms and expectations played a significant role in participants' decision not to seek support. The concept of self‐harm clashed with valued family traditions and definitions of success within their community:

Most African parents will push you towards that goal, go to school, have a good career, that's the only way you're gonna be successful. It's like a cultural thing, you know, be a Doctor, Lawyer, Engineer. Self‐harm doesn't really fit in with that. (P5)

Participants described how vulnerability was perceived as a weakness. There was a large sense of pressure to ‘keep it moving’ (P3) in the face of adversity. One participant explained that this was born from generations and generations being socialised to be strong and ‘pick myself up by the bootstraps’ (P1) and this was embodied by them (P6):

You are raised that you're strong enough to handle anything…so when life throws you lemons, like cool, lets make some lemonade, okay well these lemons are sour, cool, well how else can I see this situation in a positive way. I'm never a victim and if I'm never a victim, then why do I need help from somebody else? (P6)

Expected roles and gendered responsibilities imposed additional pressures, yet were described as important and valued within Black British communities. For some participants, these expectations created a sense of obligation to cope independently, meaning responsibility itself functioned as both a source of stress and a barrier to seeking help. One participant reflected on culturally embedded expectations of self‐reliance, noting, ‘we would rather do things ourselves than ask for help because we can do it’ *(P1)*. Similarly, another participant described anticipating a lack of understanding or support, explaining, ‘no one will give you a listening ear and no one will even support you so I tried to do everything myself’ *(P2)*. This participant's fear of being perceived as failing their expected gender role further discouraged disclosure and help‐seeking: ‘everyone may be looking at me as I failed my duty as a man.’ *(P2)*

### Promoting equity

This theme examines participants' experiences and preferences at the intersections of ethnicity, help‐seeking and meaningful community‐based support. While support preferences varied due to individual experiences, all highlighted the need for organisations to acknowledge their diversity and better consider access barriers.

#### Unaddressed hardships

A shared commonality within participants' accounts was that even the best‐intentioned community support was only as ‘good as how many of us can get to it’ (P4). Most participants noted how organisations were not visible:

I don't think I've ever seen anything about self‐harm groups or help, maybe pregnancy support or you know, get your vaccinations or things like that, I didn't necessarily see things that were like “okay this is a space where you can come and erm you can talk.” (P3)

Participants believed that visible organisations needed to better consider the systemic barriers to access faced by marginalised groups. As one participant poignantly noted, the very act of identifying and seeking help for self‐harm stems from a position of privilege:

Identifying self‐harm and mental health as issues comes from a space of privilege, in the sense of being able to slow your life down. People in minority groups that are not from the East erm don't have always the luxury of slowing down. We are just about managing to put food on the table. (P6)

Here, participants described compounding hardships, such as financial instability, which were ill‐considered within support. Participants wanted services to first consider if people had the resources to do what was recommended for things like ‘self‐care’:

A solution‐based focus hasn't been that helpful for me because I can't always use those solutions, and when I can't use a solution there is a chance I will just fall back on to self‐harm. (P1)

There was also a need among participants for professionals to acknowledge how difficult it may be for the person to be at that session:

Acknowledging the strength it takes to reach out to services to begin with. That person probably feels completely alone in that situation, they probably don't know anyone else who is doing this, who looks like them, and when it comes to services, a lot of the time the people that they are going to be interacting with don't look like them. It feels really foreign to you, takes a lot of power. (P1)

#### Person centred care – ‘we're not a monolith’

All participants highlighted that ethnicity, mental health and self‐harm are deeply intertwined, and yet are treated as three separate things, with ethnicity specifically being most unrecognised in support. This acted as a barrier to accessing what they considered meaningful support:

[My] identity plays a big role in influencing my mental health, but she [support leader] was White British and, I don't (SIGH), I tried to express this to her but it was almost dismissed, like my identity wasn't really being recognised. (P2)

For half of the participants, the lack of institutional representation was a problem due to perceptions that ‘outsider’ clinicians would not be able to fully understand their traditions and experiences of navigating discrimination. It was perceived, therefore, that a Black British therapist or leader would reduce the need to explain the intricacies of their identity and be less likely to pass judgement:

remember when my mum cut my afro‐type kinky hair…that was such a catastrophic event and led to my first suicide attempt. If a professional already understands why that's such a big deal because they are also black and they understand just how much importance hair is put on to black women within the black community, that would've been comforting to me and I wouldn't have to worry about filling them in on every single detail…. I wouldn't feel so stupid saying I wanted to die because mum cut my hair off. (P1)

On the contrary, two participants preferred a clinician from outside of the Black British community. They described how it felt easier to talk to someone who is less likely to share the same values as someone from within the Black British community:

If he has the same mindset with others in the tradition, I think he would have little sentiment for me, because he may have tried to believe them or tried to think otherwise about my thoughts. (P2)

However, while preferences varied, the shared sentiment among participants lied in their need for services to acknowledge, and not judge, their rich diversity and individual experiences.

we're not a monolith, so there will be varied experiences, varied understanding, depending on where we grew up, who we were raised by, how old we are, so not to just assume I have a particular stereotype and work in accordance with that. (P3)

#### Helpful systems

Positive experiences of community‐based services were shared by participants. Participants shared in the sentiment of enjoying being exposed to other Black British people also experiencing mental health difficulties: ‘I could finally speak about what was going on in my mind’ (P5):

I also did like a group as well, seeing other people as well and seeing their experience was so helpful. (P2)

The flexibility of an organisation was prioritised, as it was important that in times of distress, no extra pressure or expectation was placed on participants. Voluntary services allowing them to ‘come and go when I want or need’ (P2), as opposed to the expectation of complete participation was appreciated by participants:

The charities or support groups I've contacted, it was rather simple, I kind a just turned up to the support group sessions. It was great, but when it came to something like counselling where I would talk about self‐harm, I would have to go through quite a bit of paperwork. (P1)

Participants considered the best organisations to be the spaces where meaningful discussion helped them make sense of their individual experiences of mental distress and self‐harm in relation to their identity. One participant recommends working with discourses of strength to enhance utilisation:

Some cultures people need help, other cultures wanna help themselves, if you wanna help me, help me to find a way to help myself….Help them open up to where they feel like they're helping themselves, as opposed to like they're getting help….Cause if they feel like they are getting help, boom, it will be ‘I'm good, I can take care of myself (LAUGH)’. (P6)

## DISCUSSION

This study aimed to explore Black British individual's thoughts, feelings and experiences of self‐harm, and to understand their lived experience of seeking, or not seeking, community‐based support. Three themes emerged from participants. Namely, ‘complexities of self‐harm’, ‘navigating expectations’ and ‘promoting equity’.

### Complexities of self‐harm

Participants described the use of multiple, co‐occurring, yet less conventionally understood, forms of self‐harm. This reflects a current understanding of self‐harm, whereby individuals may experiment with different methods at once (Edmondson, 2013). Lending empirical support, Kapur et al. (2013) found that over 60% of participants switched to a different form of self‐harm after cutting. The inference drawn from this is that a limited understanding of the different methods of self‐harm may hinder the exploration of a person's self‐harm experience.

Self‐harm was perceived as an important, albeit potentially dangerous, regulatory mechanism in times of distress for participants. Specifically, self‐harm served as a means of managing overwhelming emotions and distressing thoughts. This is consistent with numerous studies confirming affect regulation as a primary function of self‐harm (Kuehn et al., 2022; McKenzie & Gross, 2014). Additionally, participants provided valuable insights into their competing cognitions toward self‐harm. On one hand, self‐harm was experienced as empowering and provided a sense of accomplishment. On the other, it failed to fully resolve the presenting difficulty, and for some, caused further distress.

This paradoxical relationship subscribes to the ‘benefits and barriers’ model proposed by Hooley and Franklin (2017). Within the model, self‐harm is explained to fulfil important psychological functions, such as distraction from and demonstration of distress. Meanwhile, further interpersonal and intra‐personal consequences are created from engaging in the act, such as scarring and shame that can lead to isolation and damage self‐esteem.

Relatedly, in Gray et al. (2023) more recent exploration of this phenomenon, the experience of ambivalence toward self‐harm was associated with higher levels of psychological distress when compared to those who solely wanted to avoid the behaviour. Its relevance lies in illustrating how ambivalence itself may function as a risk factor for poorer mental health and the potential perpetuation of self‐harm over time. In line with these perspectives, the addictive nature of self‐harm, as described by participants, appears crucial for understanding perpetuation, supporting how self‐harm can transition from a transient coping strategy to an entrenched habit (Worley, 2020).

### Navigating expectations

Participants' early encounters with racism and social ‘othering’ fostered struggles with identity development and sense of belonging from a young age. This aligns with research demonstrating racism's detrimental long‐term effects on mental health (Nazroo et al., 2019; Wallace et al., 2016). Self‐harm was employed as a form of punishment for lacking White attributes and as a method to ‘correct’ Black British racial features. This is a novel finding that offers important insights into the intersections of race and self‐harm. It confirms Stänicke's (2021) proposition that self‐directed pain can be experienced as deserved and represent a frustration of being restricted in life. Secondly, it supposes that services may benefit from exploring how internalised racial biases may influence self‐harm experiences.

The punishing component of self‐harm has been identified in previous literature (Klonsky, 2007). Some researchers use early Freudian formulations of guilt and self‐directed aggression to understand contemporary perspectives (Goldblatt, 2010; Parfitt, 2006). Specifically, Freud's concept of ‘moral masochism’ (Freud, 1924) suggests perceived short‐comings and guilt can manifest through self‐directed suffering. This may echo the role of self‐punishment for perceived deficiencies.

Also surfacing was how persistent stigma and intergenerational differences toward self‐harm acted as barriers to seeking help and disclosure for participants. The construction of help‐seeking attitudes has been found to be underpinned by culture, and the meanings which they assign to them (Fernando, 2014). Exposure to opinions within Black British communities that connect mental health challenges to being a ‘failure’ may increase tendencies to conceal difficulties and decide against seeking help (Kovandžić et al., 2011).

Expectations of strength also deterred participants from disclosing difficulty, and this is well documented in extant literature (Rabiee & Smith, 2013; Romero, 2000). The supposition here is that strength was regarded as an important, yet sometimes problematic, performance that placed a hard‐to‐achieve expectation on participants and perpetuated the idea that one must cope alone. Nelson et al. (2016) offers support and suggests concepts of strength may be making it more difficult for individuals to recognise and accept mental health challenges (Nelson et al., 2016).

### Promoting equity

Participants appreciated the informality afforded by community‐based support services when compared to statutory services and called for more ‘come‐and‐go’ services. During times of distress, engaging with support on their own terms was a facilitator for access. Recent research has found that a lack of flexibility in community‐based services can act as a barrier to service utilisation (Hulin et al., 2024). For marginalised groups, having autonomy and choice in one's own care is important for self‐determination and empowerment (Rwebugisa, 2020). Of course, this was only possible when participants were aware of these services. For some, there was a lack of visibility and awareness of these organisations. Concerningly, Vickers (2000) suggests that the invisibility of support can perpetuate stigma by implying that certain issues should not be discussed.

Perceived cultural naivety and the desire to be understood within one's cultural context meant that some participants preferred to be ethnically matched to their clinician. This mirrors previous literature of participant preference among Global Majority service users (Cabral & Smith, 2011) and acted as a facilitator for service access (Tabassum et al., 2000). When ethnicity is shared, services are perceived as more approachable (Li et al., 1999), better supported with language barriers (McClay et al., 2013), and reduce the stigma related to getting help through representation (Singla et al., 2014). This illustrates the importance of establishing trust within the therapeutic relationship (Kelly & Strupp, 1992).

Concerns around representation in services can also be understood within the context of workforce composition. Data from the NHS Workforce Race Equality Standard (NHS England, 2025) consistently highlight underrepresentation of racially minoritised groups, particularly at senior and leadership levels. This lack of representation may shape service users' expectations of whether their cultural and racial identities will be understood or valued within care settings. For some participants, such as those describing experiences around hair and appearance, representation mattered because it reduced the need to explain or justify aspects of their identity, fostering a greater sense of cultural and racial safety.

An important development was found in how some participants preferred not to be ethnically matched to a clinician. Here, participants described feeling more comfortable with perspectives held from outside of the Black British community. This preference could demonstrate Green et al. (2019) point that fears of community stigma can breed distrust and be generalised to healthcare systems. It serves as a powerful illustration of the need for staff to recognise and respect service‐user culture and to facilitate discussions of challenges that may be particularly stigmatised within their own community.

Finally, some participants felt that recognising one's mental health challenges required privilege they did not have due to financial and time pressures. This corroborates with Brown et al. (2021) supposition that living at the intersections of oppression, daily realities of poverty, racism, and other stressors leave little capacity to prioritise mental wellness.

These accounts are situated within a broader socio‐economic context. Data reported by the UK Government (GOV.UK, 2025), drawing on Office for National Statistics sources, indicate that long‐standing patterns of economic hardship continue in the United Kingdom, disproportionately affecting Black ethnic communities compared with White populations. This pattern has remained stable across generations, reflecting enduring structural inequalities rather than transient economic disadvantage. Such persistent socio‐economic inequity provides important context for participants' accounts of help‐seeking as a privilege, reinforcing how financial precarity and time constraints can limit access to mental health support. Kovandžić et al. (2011) concurred while anti‐stigmatic movements and increased information of services can facilitate access, a critical obstacle to equitable access lies in the availability of services that are culturally attuned and experienced as appropriate by underserved groups.

Without broader efforts to dismantle systemic inequities at their roots, even the most well‐meaning programmes will struggle to reach those most in need.

### Strengths, limitations and future directions

The study addresses literature gaps on the intersections of self‐harm and race/ethnicity, and offers perspectives not previously found in the literature. Importantly, this study provided participants an opportunity to express their truth. The researcher was thanked for providing a space for their voice to be heard, and one stated the research topic itself felt ‘like an ally’.

Given that cultural mistrust can affect participation (American Psychological Association, 2023b) the researcher's overt ‘membership’ to being Black British, may have facilitated a greater sense of safety for participants in voicing their experiences. This likely generated an openness within the interviews, as opposed to consciously filtering responses to avoid feeling uncomfortable or offensive. That said, the researcher acknowledges how this could be mitigated due to the researchers position of being a mental health professional. Conversely, keeping true to the findings of this study, some participants might have withheld information through fear of being judged by another Black British individual.

A limitation of the study is that it did not distinguish between the different types of community‐based services from which the participants sought help in the analysis. The participants received support from various community‐based organisations; however, these services may not have specialised in self‐harm issues or culturally specific support for Black British communities. This limits the depth of the findings. Future research should distinguish between tailored and general services to better understand how well these services align with population needs and influence perceptions and engagement.

To ensure the research aligns with the needs, preferences, and priorities of those directly affected by the research topic (Jennings et al., 2018), the study invited a PPI contributor. However, engaging a PPI collaborator after the protocol and tools had already been designed does not constitute true co‐design and partnership in the research process. The researcher may have denied the study an opportunity to gain an even richer cultural understanding had it practised higher levels of collaboration. Future research should practise the truest forms of collaboration when designing research to maximise impact and empowerment.

While this study does not aim to examine intersectional influences on self‐harm or help‐seeking, existing research has highlighted gender differences in patterns of self‐harm among Black British populations, particularly when comparing Black males and females with other ethnic groups (Cooper et al., 2010; Farooq et al., 2021). This suggests that future research may benefit from exploring self‐harm and help‐seeking separately across different gender identities to better understand how gendered expectations and social roles shape these experiences. Additionally, the term ‘Black British’ seeks to acknowledge African and Caribbean heritages; however, the term risks ignoring intra‐ethnic diversity. This includes variations in cultural practises, identities, migration histories and values which may shape experiences of distress and pathways to care in nuanced ways.

### Implications

Participants' experiences were uniquely situated within a specific context, and do not represent the wider population, or even all Black British individuals who self‐harm. However, facilitating meaningful change requires understanding common patterns of meaning to inform practise and service development (Tracy, 2010). The findings highlight several implications for practitioners and community‐based services working with Black British individuals who self‐harm.

First, support services should recognise the diverse manifestations of self‐harm beyond stereotypical presentations such as cutting. Without acknowledging different forms of self‐harm and their personal relevance, practitioners risk engaging in a narrow exploration of service‐user experience and overlooking important avenues for therapeutic change (Hetrick et al., 2020; Kothgassner et al., 2020). As illustrated in this study, participants often held ambivalent and conflicting relationships with self‐harm, simultaneously wanting to and not wanting to engage in the behaviour. These conflicts may be crucial to understanding escalation and repetition (Gill et al., 2023). Services should therefore protect space and time for open discussions about self‐harm itself, rather than framing it solely as a symptom of other mental health difficulties, which may reinforce beliefs that it should not be spoken about.

Secondly, the findings challenge dominant narratives that position Black populations as ‘hard to reach’ (Davies et al., 2020). Such language risks perpetuating stereotypes and deflecting attention away from structural barriers and inequalities faced by people of the Global Majority. Access, then, is best considered as a dynamic relationship between the service *and* the characteristics of the individual in which the service is designed to support. Systemic exclusion may be perpetuated by a lack of culturally relevant psychological therapies despite professional guidelines emphasising the importance of cultural sensitivity and responsiveness (The British Psychological Society, 2017). However, within the UK problems persist with evidence from the NHS Race & Health Observatory showing that Black and minoritised ethnic groups face lower access rates, longer wait times, and poorer outcomes in IAPT services than White British groups (NHSRHO, 2022). These findings reinforce our participants' accounts of ongoing systemic barriers within the programme. Race, ethnicity, and culture should therefore be considered during assessment, integrated into formulation, and inform culturally adaptive interventions to support Black British service users in navigating societal influences and improving long‐term outcomes (NICE, 2022). In line with this, practitioners are encouraged to reflect on whether therapeutic approaches shaped predominantly by Eurocentric norms adequately address the diverse worldviews, preferences, and lived realities of Black British service users. Practitioners should ensure that wellbeing is defined by the service and consider if models are fully attentive to cultural and systemic influences (Stubbe, 2020). Services may benefit from exploring if the recommended coping strategies, emotion regulation techniques, and safety plans are realistic and achievable within the social, economic, and time‐related constraints faced by individuals (Fonagy & Luyten, 2021; NICE, 2022). Greater attention to cultural and systemic influences may support more meaningful engagement and reduce reliance on strategies that inadvertently place responsibility solely on the individual.

The specific cultural and historical contexts shaping Black British people's experience of self‐harm also warrant dedicated enquiry. Given that Black British identity is constructed within a postcolonial legacy of slavery and societal racism (Mariska et al., 2019), practitioners should remain alert to the forms of discrimination and environmental oppression that may be impacting service users both within and outside therapeutic spaces (NICE, 2022). Discourses of strength and stigma may discourage vulnerability and lead individuals to avoid discussing even the most relevant aspects of their distress (Akoury et al., 2019). Practitioners are therefore encouraged to approach these topics with sensitivity and curiosity, initiating conversations rather than waiting for disclosure.

Therapeutic approaches that resist shame and reframe vulnerability as a form of strength may be particularly beneficial (Graham et al., 2013). Community‐based services may benefit from working within, rather than against, existing discourses of strength by adopting approaches that foreground individuals' inherent resources and capacities (Romero, 2000). In doing so, services may support service users to develop a greater sense of ownership over their recovery, which has been associated with improved engagement and utilisation (Ryan et al., 2010). Recent guidance on community mental health services further supports person‐centred and rights‐based approaches that attend to identity, community relationships, and broader aspirations, moving away from one‐size‐fits‐all models of care (World Health Organisation, 2021).

The findings of this study do not completely concur with previous research suggesting that ethnic matching is universally preferred. Some participants described a preference not to work with Black British practitioners due to fears of being, metaphorically, ‘too close’ and of encountering shared stigmatic views from within their own community. Conversely, other participants expressed a preference for ethnically matched practitioners, driven by concerns that professionals from other cultural backgrounds may be ‘too far away’ to fully understand self‐harm in relation to their identity. This variability in preferences underscores the importance of cultural competence for practitioners, regardless of their race or ethnicity. No single approach will suit all individuals, and service users should feel empowered to receive care on their own terms (NICE, 2022). Practitioners should practise cultural humility by acknowledging the limitations of their own experiences and actively work to understand unique worldviews without judgement (Stubbe, 2020). This demonstrates a need for ongoing reflexivity and training for both lay and professional workers within community organisations.

Finally, improving awareness and accessibility of services may require proactive outreach strategies tailored to Black British populations (Hanif, 2023). This may include intentional relationship‐building within cultural communities and community organisations to support trust, visibility and engagement with available support (Kalathil et al., 2011).

## CONCLUSION

This study provided important insights into the construction and experience of self‐harm and help‐seeking among Black British individuals. Internalised racism played a role in the onset of self‐harm which served as a punishment, refuge from distress and an empowering experience for some participants. It also invited further hardship and feelings of shame and guilt. Participants' construction of self‐harm occurred in the context of societal norms and traditional values within their communities. Here, recognising and seeking help for mental health challenges was regarded as a privilege some did not have. This experience was further compounded by stigma and expectations of strength which discouraged vulnerability and help‐seeking. Community‐based services were valued when they were visible. However, participants called for greater acknowledgement of their intersecting identities and experiences of self‐harm. Services should promote culturally attuned self‐harm management strategies that consider individuals' fears of being misunderstood and socioeconomic realities.

## AUTHOR CONTRIBUTIONS

**Olivia Alleyne:** Conceptualization; investigation; writing – original draft; methodology; project administration; writing – review and editing. **Vyv Huddy:** Conceptualization; investigation; methodology; writing – review and editing; supervision.

## CONFLICT OF INTEREST STATEMENT

The authors declare no conflicts of interest.

## Data Availability

Research data are not shared.

## Footnotes

^1^
The author recognises that concepts of ‘race’ and ‘ethnicity’ are social constructs (Wijekoon & Peter, 2022). However, acknowledging how they shape lived experience is crucial for this study when considering the diversity within Black British communities. In keeping with the Journal Article of Reporting Standards for Race, Ethnicity and Culture (JARS‐REC; American Psychological Association, 2023a), these concepts are employed, yet defined, to avoid amalgamation and reflect intragroup differences. Thus, ‘race’ refers to groupings based on physical characteristics, such as skin colour. ‘Ethnicity’ refers to a shared cultural identity through factors such as heritage, language, and beliefs (American Psychological Association, 2023a, 2023b).

^2^
The ‘third sector’ is an umbrella term used to refer to value‐driven, non‐governmental and non‐profit groups that work at the community‐level to serve societal needs. Examples include charities, volunteer groups and social enterprises (National Audit Office, 2023). For this review, the term ‘community‐based support’ is employed throughout to illustrate the level at which these organisations exist.

^3^
The term ‘People of the Global Majority’ reframes how ‘minority’ populations constitute the majority of the world's population.

## References

[papt70063-bib-0001] Afuwape, S. A. , Craig, T. K. J. , Harris, T. , Clarke, M. , Flood, A. , Olajide, D. , Cole, E. , Leese, M. , McCrone, P. , & Thornicroft, G. (2010). The cares of life project (COLP): An exploratory randomised controlled trial of a community‐based intervention for black people with common mental disorder. Journal of Affective Disorders, 127(1–3), 370–374. 10.1016/j.jad.2010.05.017 20547421

[papt70063-bib-0002] Akoury, L. M. , Warren, C. S. , & Culbert, K. M. (2019). Disordered eating in Asian American women: Sociocultural and culture‐specific predictors. Frontiers in Psychology, 10, 1950. 10.3389/fpsyg.2019.01950 PMC6737071 31551855

[papt70063-bib-0003] Al‐Sharifi, A. , Krynicki, C. R. , & Upthegrove, R. (2015). Self‐harm and ethnicity: A systematic review. International Journal of Social Psychiatry, 61(6), 600–612. 10.1177/0020764015573085 25783961

[papt70063-bib-0004] American Psychological Association . (2023a). JARS – Race, ethnicity, and culture. https://apastyle.apa.org/jars/rec‐table‐1.pdf

[papt70063-bib-0005] American Psychological Association . (2023b). Race and ethnicity. https://www.apa.org/topics/race‐ethnicity

[papt70063-bib-0006] Bamford, J. , Klabbers, G. , Curran, E. , Rosato, M. , & Leavey, G. (2020). Social capital and mental health among black and minority ethnic groups in the UK. Journal of Immigrant and Minority Health, 23(3), 502–510. 10.1007/s10903-020-01043-0 PMC8068661 32623610

[papt70063-bib-0008] Baskin, C. , Zijlstra, G. , McGrath, M. , Lee, C. , Duncan, F. H. , Oliver, E. J. , Osborn, D. , Dykxhoorn, J. , Kaner, E. F. , LaFortune, L. , Walters, K. R. , Kirkbride, J. , & Gnani, S. (2021). Community‐centred interventions for improving public mental health among adults from ethnic minority populations in the UK: A scoping review. BMJ Open, 11(4), e041102. 10.1136/bmjopen-2020-041102

[papt70063-bib-0009] Beauboeuf‐Lafontant, T. (2008). Listening past the lies that make us sick: A voice‐centered analysis of strength and depression among black women. Qualitative Sociology, 31(4), 391–406. 10.1007/s11133-008-9113-1

[papt70063-bib-0010] Bentall, D. P. (1999). The medicalisation of misery: A critical realist analysis of the concept of depression. Journal of Mental Health, 8(3), 261–274. 10.1080/09638239917427

[papt70063-bib-0013] Brown, Y. , Brown, G. , & Goodman, S. (2021). *Black men, mental health and oppression: What do we learn when we listen to black men's voices?* (thesis). *Black men, mental health and oppression: what do we learn when we listen to Black men's voices?* Coventry University, Great Britain.

[papt70063-bib-0014] Burgess, R. A. , Kanu, N. , Matthews, T. , Mukotekwa, O. , Smith‐Gul, A. , Yusuf, I. , Lamptey, I. , McCauley, N. , Wilson, R. , Pirisola, M. , & Gul, M. (2022). Exploring experiences and impact of the COVID‐19 pandemic on young racially minoritised people in the United Kingdom: A qualitative study. PLoS One, 17(5), e0266504. 10.1371/journal.pone.0266504 PMC9067664 35507595

[papt70063-bib-0015] Cabral, R. R. , & Smith, T. B. (2011). Racial/ethnic matching of clients and therapists in mental health services: A meta‐analytic review of preferences, perceptions, and outcomes. Journal of Counseling Psychology, 58(4), 537–554. 10.1037/a0025266 21875181

[papt70063-bib-0016] Calò, F. , Montgomery, T. , & Baglioni, S. (2021). Marginal players? The third sector and employability services for migrants, refugees and asylum seekers in the UK. Voluntas: International Journal of Voluntary and Nonprofit Organizations, 33(5), 872–885. 10.1007/s11266-020-00306-6 PMC7781423 33424139

[papt70063-bib-0017] Chakraborty, A. T. , McKenzie, K. J. , Hajat, S. , & Stansfeld, S. A. (2010). Racism, mental illness and social support in the UK. Social Psychiatry and Psychiatric Epidemiology, 45(12), 1115–1124. 10.1007/s00127-009-0156-8 19847373

[papt70063-bib-0018] Cooper, J. , Murphy, E. , Webb, R. , Hawton, K. , Bergen, H. , Waters, K. , & Kapur, N. (2010). Ethnic differences in self‐harm, rates, characteristics and service provision: Three‐city cohort study. British Journal of Psychiatry, 197(3), 212–218. 10.1192/bjp.bp.109.072637 20807966

[papt70063-bib-0127] Davies, J. , McKenna, M. , Bayley, J. , Denner, K. , & Young, H. (2020). Using engagement in sustainable construction to improve mental health and social connection in disadvantaged and hard to reach groups: A new green care approach. Journal of Mental Health, 29(3), 350–357. 10.1080/09638237.2020.1714001 32036713

[papt70063-bib-0019] Edge, D. (2007). Ethnicity, psychosocial risk, and perinatal depression—A comparative study among inner‐city women in the United Kingdom. Journal of Psychosomatic Research, 63(3), 291–295. 10.1016/j.jpsychores.2007.02.013 17719367

[papt70063-bib-0020] Edmondson, A. J. (2013). *Listening with your eyes: Using pictures and words to explore self‐harm* (thesis). *Listening with your eyes: using pictures and words to explore self‐harm* . University of Leeds, Great Britain.

[papt70063-bib-0021] Edmondson, A. J. , Brennan, C. A. , & House, A. O. (2016). Non‐suicidal reasons for self‐harm: A systematic review of self‐reported accounts. Journal of Affective Disorders, 191, 109–117. 10.1016/j.jad.2015.11.043 26655120

[papt70063-bib-0022] Elliott, R. , Fischer, C. T. , & Rennie, D. L. (1999). Evolving guidelines for publication of qualitative research studies in psychology and related fields. The British Journal of Clinical Psychology, 38(3), 215–229. 10.1348/014466599162782 10532145

[papt70063-bib-0023] Farooq, B. , Clements, C. , Hawton, K. , Geulayov, G. , Casey, D. , Waters, K. , Ness, J. , Patel, A. , Kelly, S. , Townsend, E. , Appleby, L. , & Kapur, N. (2021). Self‐harm in children and adolescents by ethnic group: An observational cohort study from the multicentre study of self‐harm in England. The Lancet Child & Adolescent Health, 5(11), 782–791. 10.1016/s2352-4642(21)00239-x PMC9766885 34555352

[papt70063-bib-0024] Favazza, A. R. (1996). Bodies under siege: Self‐mutilation and body modification in culture and psychiatry. Johns Hopkins University Press.

[papt70063-bib-0125] Fernando, S. (2014). Mental health worldwide: Culture, globalization and development. Palgrave Macmillan.

[papt70063-bib-0025] Fonagy, P. , & Luyten, P. (2021). Socioeconomic and sociocultural factors affecting access to psychotherapies: The way forward. World Psychiatry, 20(3), 315–316. 10.1002/wps.20911 PMC8429320 34505381

[papt70063-bib-0026] Freud, S. (1924). The Economic Problem of Masochism. The Psychoanalytic Review, 19, 157–172.

[papt70063-bib-0027] Geronimus, A. T. (1992). The weathering hypothesis and the health of African‐American women and infants: Evidence and speculations. Ethnicity & Disease, 2(3), 207–221. 1467758

[papt70063-bib-0028] Geulayov, G. , Casey, D. , McDonald, K. C. , Foster, P. , Pritchard, K. , Wells, C. , Clements, C. , Kapur, N. , Ness, J. , Waters, K. , & Hawton, K. (2018). Incidence of suicide, hospital‐presenting non‐fatal self‐harm, and community‐occurring non‐fatal self‐harm in adolescents in England (the iceberg model of self‐harm): A retrospective study. The Lancet Psychiatry, 5(2), 167–174. 10.1016/s2215-0366(17)30478-9 29246453

[papt70063-bib-0029] Gill, G. , Singh, G. , Mitra, S. , Tran, K. , & Korenis, P. (2023). I burn myself to get high. The Primary Care Companion for CNS Disorders, 25(3), 329. 10.4088/pcc.22cr03329 37230062

[papt70063-bib-0031] Goldblatt, M. J. (2010). Suicide and masochism: The evolving relationship between guilt, suffering, self‐attack and suicide. Psychoanalytic Psychotherapy, 24(2), 93–100. 10.1080/02668731003707733

[papt70063-bib-0032] GOV.UK . (2025). People in low‐income households Ethnicity facts and figures. https://www.ethnicity‐facts‐figures.service.gov.uk/work‐pay‐and‐benefits/pay‐and‐income/people‐in‐low‐income‐households/latest/

[papt70063-bib-0033] Graham, J. R. , Sorenson, S. , & Hayes‐Skelton, S. A. (2013). Enhancing the cultural sensitivity of cognitive behavioral interventions for anxiety in diverse populations. The Behavior Therapist, 36(5), 101–108. PMC4225557 25392598

[papt70063-bib-0034] Gray, D. E. (2018). Doing research in the real world. Sage Publications.

[papt70063-bib-0035] Gray, N. , Uren, H. , Pemberton, E. , & Boyes, M. (2023). Profiling ambivalence in the context of nonsuicidal self‐injury. Journal of Clinical Psychology, 79(8), 1699–1712. 10.1002/jclp.23494 PMC10952785 36840691

[papt70063-bib-0036] Green, H. D. , Weeks, M. R. , Berman, M. , Salvi, A. , Gonzalez, R. , Rohena, L. , Ferguson, A. , & Li, J. (2019). The impact of perceptions of community stigma on utilization of HIV care services. Journal of Racial and Ethnic Health Disparities, 7(2), 383–391. 10.1007/s40615-019-00667-9 PMC7067618 31808137

[papt70063-bib-0037] Halvorsrud, K. , Nazroo, J. , Otis, M. , Brown Hajdukova, E. , & Bhui, K. (2019). Ethnic inequalities in the incidence of diagnosis of severe mental illness in England: A systematic review and new meta‐analyses for non‐affective and affective psychoses. Social Psychiatry and Psychiatric Epidemiology, 54(11), 1311–1323. 10.1007/s00127-019-01758-y 31482194

[papt70063-bib-0038] Hanif, B. (2023). How to create a community outreach strategy? GlueUP. https://www.glueup.com/blog/community‐outreach‐strategy#:~:text=A%20proactive%20community%20outreach%20strategy,add%20value%20to%20the%20community.

[papt70063-bib-0039] Hauser, H. J. (2023). More than my experience: An argument for critical realism in person‐centred psychotherapy. Person‐Centered & Experiential Psychotherapies, 3, 1–18. 10.1080/14779757.2023.2295528

[papt70063-bib-0042] Hetrick, S. , Subasinghe, A. , Anglin, K. , Hart, L. , Morgan, A. , & Robinson, J. (2020). Understanding the needs of young people who engage in self‐harm: A qualitative investigation. Frontiers in Psychology, 10, 2916. 10.3389/fpsyg.2019.02916 PMC6968776 31998182

[papt70063-bib-0043] Hooley, J. M. , & Franklin, J. C. (2017). Why do people hurt themselves? A new conceptual model of nonsuicidal self‐injury. Clinical Psychological Science, 6(3), 428–451. 10.1177/2167702617745641

[papt70063-bib-0044] Hulin, J. , Huddy, V. , Oliver, P. , Marshall, J. , Mohindra, A. , Delaney, B. , & Mitchell, C. (2024). Experiences of support for people who access voluntary, community and social Enterprise (VCSE) organisations for self‐harm: A qualitative study with stakeholder feedback. BMC Public Health, 24(1), 1059. 10.1186/s12889-024-18455-4 PMC11020776 38627716

[papt70063-bib-0047] Jennings, H. , Slade, M. , Bates, P. , Munday, E. , & Toney, R. (2018). Best practice framework for patient and public involvement (PPI) in collaborative data analysis of qualitative mental health research: Methodology development and refinement. BMC Psychiatry, 18(1), 213. 10.1186/s12888-018-1794-8 PMC6022311 29954373

[papt70063-bib-0048] Jidong, D. E. , Husain, N. , Bailey, A. , & Okoloba, M. M. (2024). Interventions for self‐harm and suicidal ideation in Africa: A systematic review. Archives of Suicide Research, 29(1), 1–25. 10.1080/13811118.2024.2316168 38506246

[papt70063-bib-0126] Kalathil, J. , Collier, B. , Bhakta, R. , Daniel, O. , Joseph, D. , & Trivedi, P. (2011). Recovery and resilience: African, African Caribbean and South Asian Women's stories of recovering from mental distress. Mental Health Foundation.

[papt70063-bib-0049] Kapur, N. , Cooper, J. , O'Connor, R. C. , & Hawton, K. (2013). Non‐suicidal self‐injury v. attempted suicide: New diagnosis or false dichotomy? British Journal of Psychiatry, 202(5), 326–328. 10.1192/bjp.bp.112.116111 23637107

[papt70063-bib-0050] Keating, F. , & Robertson, D. (2002). Breaking the circles of fear: A review of mental health services to African and Caribbean communities. PsycEXTRA Dataset. 10.1037/e427032008-011

[papt70063-bib-0051] Keating, F. , & Robertson, D. (2004). Fear, black people and mental illness: A vicious circle? Health & Social Care in the Community, 12(5), 439–447. 10.1111/j.1365-2524.2004.00506.x 15373823

[papt70063-bib-0052] Kelly, T. A. , & Strupp, H. H. (1992). Patient and therapist values in psychotherapy: Perceived changes, assimilation, similarity, and outcome. Journal of Consulting and Clinical Psychology, 60(1), 34–40. 10.1037//0022-006x.60.1.34 1556283

[papt70063-bib-0053] Klonsky, E. D. (2007). The functions of deliberate self‐injury: A review of the evidence. Clinical Psychology Review, 27(2), 226–239. 10.1016/j.cpr.2006.08.002 17014942

[papt70063-bib-0054] Kothgassner, O. D. , Robinson, K. , Goreis, A. , Ougrin, D. , & Plener, P. L. (2020). Does treatment method matter? A meta‐analysis of the past 20 years of research on therapeutic interventions for self‐harm and suicidal ideation in adolescents. Borderline Personality Disorder and Emotion Dysregulation, 7(1), 9. 10.1186/s40479-020-00123-9 PMC7216729 32426138

[papt70063-bib-0055] Kovandžić, M. , Chew‐Graham, C. , Reeve, J. , Edwards, S. , Peters, S. , Edge, D. , Aseem, S. , Gask, L. , & Dowrick, C. (2011). Access to primary mental health care for hard‐to‐reach groups: From ‘silent suffering’ to ‘making it work’. Social Science and Medicine, 72(5), 763–772. 10.1016/j.socscimed.2010.11.027 21272968

[papt70063-bib-0056] Kuehn, K. S. , Dora, J. , Harned, M. S. , Foster, K. T. , Song, F. , Smith, M. R. , & King, K. M. (2022). A meta‐analysis on the affect regulation function of real‐time self‐injurious thoughts and behaviours. Nature Human Behaviour, 6(7), 964–974. 10.1038/s41562-022-01340-8 PMC9329197 35484208

[papt70063-bib-0057] Larkin, M. , Watts, S. , & Clifton, E. (2006). Giving voice and making sense in interpretative phenomenological analysis. Qualitative Research in Psychology, 3(2), 102–120. 10.1191/1478088706qp062oa

[papt70063-bib-0058] Levesque, J.‐F. , Harris, M. F. , & Russell, G. (2013). Patient‐centred access to health care: Conceptualising access at the interface of health systems and populations. International Journal for Equity in Health, 12(1), 18. 10.1186/1475-9276-12-18 PMC3610159 23496984

[papt70063-bib-0059] Li, P.‐L. , Logan, S. , Yee, L. , & Ng, S. (1999). Barriers to meeting the mental health needs of the Chinese community. Journal of Public Health, 21(1), 74–80. 10.1093/pubmed/21.1.74 10321864

[papt70063-bib-0060] Lloyd‐Richardson, E. E. , Perrine, N. , Dierker, L. , & Kelley, M. L. (2007). Characteristics and functions of non‐suicidal self‐injury in a community sample of adolescents. Psychological Medicine, 37(8), 1183–1192. 10.1017/s003329170700027x PMC2538378 17349105

[papt70063-bib-0061] Madill, A. , Jordan, A. , & Shirley, C. (2000). Objectivity and reliability in qualitative analysis: Realist, contextualist and radical constructionist epistemologies. British Journal of Psychology, 91(1), 1–20. 10.1348/000712600161646 10717768

[papt70063-bib-0062] Mangalore, R. , & Knapp, M. (2011). Income‐related inequalities in common mental disorders among ethnic minorities in England. Social Psychiatry and Psychiatric Epidemiology, 47(3), 351–359. 10.1007/s00127-011-0345-0 21293847

[papt70063-bib-0063] Mariska, P. , Habibi, H. , & Farisi, A. (2019). Cultural identity of colonialism: Traumatic effects of slavery and racism. Cultura Interpreta, 9(3), 117–124.

[papt70063-bib-0065] McClay, C.‐A. , Morrison, J. , McConnachie, A. , & Williams, C. (2013). A community‐based group‐guided self‐help intervention for low mood and stress: Study protocol for a randomized controlled trial. Trials, 14(1), 392. 10.1186/1745-6215-14-392 PMC4225512 24252475

[papt70063-bib-0066] McIntosh, M. J. , & Morse, J. M. (2015). Situating and constructing diversity in semi‐structured interviews. Global Qualitative Nursing Research, 2, 233339361559767. 10.1177/2333393615597674 PMC5342650 28462313

[papt70063-bib-0067] McKenzie, K. C. , & Gross, J. J. (2014). Nonsuicidal self‐injury: An emotion regulation perspective. Psychopathology, 47(4), 207–219. 10.1159/000358097 24526099

[papt70063-bib-0068] Mclean, C. , Campbell, C. , & Cornish, F. (2003). African–Caribbean interactions with mental health services in the UK: Experiences and expectations of exclusion as (re)productive of health inequalities. Social Science and Medicine, 56(3), 657–669. 10.1016/s0277-9536(02)00063-1 12570981

[papt70063-bib-0069] McManus, S. , Gunnell, D. , Cooper, C. , Bebbington, P. E. , Howard, L. M. , Brugha, T. , Jenkins, R. , Hassiotis, A. , Weich, S. , & Appleby, L. (2019). Prevalence of non‐suicidal self‐harm and service contact in England, 2000–14: Repeated cross‐sectional surveys of the general population. The Lancet Psychiatry, 6(7), 573–581. 10.1016/s2215-0366(19)30188-9 PMC7646286 31175059

[papt70063-bib-0070] Memon, A. , Taylor, K. , Mohebati, L. , Sundin, J. , Cooper, M. , Scanlon, T. , & de Visser, R. (2016). Perceived barriers to accessing mental health services among black and minority ethnic (BME) communities: A qualitative study in Southeast England. BMJ Open, 6(11), e012337. 10.1136/bmjopen-2016-012337 PMC5128839 27852712

[papt70063-bib-0071] Morris, R. M. , Sellwood, W. , Edge, D. , Colling, C. , Stewart, R. , Cupitt, C. , & Das‐Munshi, J. (2020). Ethnicity and impact on the receipt of cognitive–behavioural therapy in people with psychosis or bipolar disorder: An english cohort study. BMJ Open, 10(12), 136. 10.1136/bmjopen-2019-0349136 PMC7745324 33323425

[papt70063-bib-0072] Mughal, F. , Burton, F. M. , Fletcher, H. , Lascelles, K. , O'Connor, R. C. , Rae, S. , Thomson, A. B. , & Kapur, N. (2023). New guidance for self‐harm: An opportunity not to be missed. The British Journal of Psychiatry, 223(5), 501–503. 10.1192/bjp.2023.113 PMC7615272 37642173

[papt70063-bib-0073] National Audit Office . (2023). Successful commissioning toolkit what are third sector organisations and their benefits for Commissioners? NAO.org. https://www.nao.org.uk/successful‐commissioning/introduction/what‐are‐civil‐society‐organisations‐and‐their‐benefits‐for‐commissioners/

[papt70063-bib-0074] National Institute for Health and Care Excellence . (2022). Self‐harm: assessment, management and preventing recurrence. NICE. https://www.nice.org.uk/guidance/ng225/resources/selfharm‐assessment‐management‐and‐preventing‐recurrence‐pdf‐66143837346757 36595613

[papt70063-bib-0075] National Institute for Health and Clinical Excellence . (2011). Self‐harm in over 8s: Long‐Term Management: Guidance. NICE. https://www.nice.org.uk/guidance/cg133 31891461

[papt70063-bib-0077] National Institute of Clinical Excellence . (2022). Recommendations: Self‐harm: Assessment, management and preventing recurrence: Guidance. NICE. https://www.nice.org.uk/guidance/ng225/chapter/recommendations 36595613

[papt70063-bib-0078] Nazroo, J. Y. , Bhui, K. S. , & Rhodes, J. (2019). Where next for understanding race/ethnic inequalities in severe mental illness? Structural, interpersonal and institutional racism. Sociology of Health & Illness, 42(2), 262–276. 10.1111/1467-9566.13001 PMC7028120 31562655

[papt70063-bib-0081] Nelson, T. , Cardemil, E. V. , & Adeoye, C. T. (2016). Rethinking strength. Psychology of Women Quarterly, 40(4), 551–563. 10.1177/0361684316646716

[papt70063-bib-0082] NHS England . (2025). NHS Workforce Race Equality Standard 2024 data analysis report for NHS trusts. https://www.england.nhs.uk/long‐read/nhs‐workforce‐race‐equality‐standard‐wres‐2024‐data‐analysis‐report‐for‐nhs‐trusts/

[papt70063-bib-0083] NHS Race and Health Observatory . (2022). Ethnic inequalities in improving access to psychological therapies (IAPT): A rapid evidence review. https://www.nhsrho.org/research/ethnic‐inequalities‐in‐improving‐access‐to‐psychological‐therapies‐iapt/

[papt70063-bib-0084] Office for Health Improvement and Disparities . (2024). Public Health Profiles. GOV.UK. https://fingertips.phe.org.uk/search/self%20harm

[papt70063-bib-0085] Olmos‐Vega, F. M. , Stalmeijer, R. E. , Varpio, L. , & Kahlke, R. (2023). A practical guide to reflexivity in qualitative research: Amee guide no. 149. Medical Teacher, 45(3), 241–251. 10.1080/0142159x.2022.2057287 35389310

[papt70063-bib-0086] Orbe, M. P. (2000). Centralizing diverse racial/ethnic voices in scholarly research: The value of phenomenological inquiry. International Journal of Intercultural Relations, 24(5), 603–621. 10.1016/s0147-1767(00)00019-5

[papt70063-bib-0087] O'Shea, R. , House, A. , & Bryant, L. (2019). The functions of self‐harm: A Q‐methodology study (thesis). The functions of self‐harm: a Q‐methodology study. University of Leeds.

[papt70063-bib-0089] Parfitt, A. (2006). On aggression turned against the self. Psychoanalytic Psychotherapy, 19(2), 160–173. 10.1080/02668730500115127

[papt70063-bib-0090] Parks, A. K. , & Hayman, L. L. (2024). Unveiling the strong black woman schema—Evolution and impact: A systematic review. Clinical Nursing Research, 33, 395–404. 10.1177/10547738241234425 38439544

[papt70063-bib-0092] Rabiee, F. , & Smith, P. (2013). Being understood, being respected: An evaluation of mental health service provision from service providers and users' perspectives in Birmingham, UK. International Journal of Mental Health Promotion, 15(3), 162–177.

[papt70063-bib-0093] Rabiee, F. , & Smith, P. (2014). Understanding mental health and experience of accessing services among African and African Caribbean service users and carers in Birmingham, UK. Diversity and Equality in Health and Care, 11, 125–134.

[papt70063-bib-0094] Rehman, Z. , Lopes, B. , & Jaspal, R. (2020). Predicting self‐harm in an ethnically diverse sample of lesbian, gay and bisexual people in the United Kingdom. International Journal of Social Psychiatry, 66(4), 349–360. 10.1177/0020764020908889 PMC7488821 32122180

[papt70063-bib-0095] Romero, R. E. (2000). The icon of the strong black woman: The paradox of strength. In Psychotherapy with African American women (pp. 225–238). The Guilford Press.

[papt70063-bib-0096] Rwebugisa, L. (2020). *The Empowerment Process: An Examination of the Development of Personal (Psychological) and Interpersonal Empowerment: A Qualitative Case Study Research Using Self‐Determination Theory* (thesis). ProQuest Dissertations & Theses.

[papt70063-bib-0097] Ryan, R. M. , Lynch, M. F. , Vansteenkiste, M. , & Deci, E. L. (2010). Motivation and autonomy in counseling, psychotherapy, and behavior change: A look at theory and practice 1ψ7. The Counseling Psychologist, 39(2), 193–260. 10.1177/0011000009359313

[papt70063-bib-0099] Scoliers, G. , Portzky, G. , Madge, N. , Hewitt, A. , Hawton, K. , Ystgaard, M. , Arensman, E. , De Leo, D. , Fekete, S. , & van Heeringen, K. (2008). Reasons for adolescent deliberate self‐harm: A cry of pain and/or a cry for help? Social Psychiatry and Psychiatric Epidemiology, 44(8), 601–607. 10.1007/s00127-008-0469-z 19023507

[papt70063-bib-0100] Simopoulou, Z. , & Chandler, A. (2020). Self‐harm as an attempt at self‐care. European Journal for Qualitative Research in Psychotherapy, 10, 110–120.

[papt70063-bib-0101] Singla, D. , Lazarus, A. , Atif, N. , Sikander, S. , Bhatia, U. , Ahmad, I. , Nisar, A. , Khan, S. , Fuhr, D. , Patel, V. , & Rahman, A. (2014). “Someone like us”: Delivering maternal mental health through peers in two south Asian contexts. Journal of Affective Disorders, 168, 452–458. 10.1016/j.jad.2014.07.017 PMC4157588 25113958

[papt70063-bib-0102] Sisley, E. J. , Hutton, J. M. , Louise Goodbody, C. , & Brown, J. S. L. (2011). An interpretative phenomenological analysis of African Caribbean Women's experiences and management of emotional distress. Health & Social Care in the Community, 19(4), 392–402. 10.1111/j.1365-2524.2010.00986.x 21314849

[papt70063-bib-0103] Smart, A. , & Harrison, E. (2017). The under‐representation of minority ethnic groups in UK medical research. Ethnicity & Health, 22(1), 65–82. 10.1080/13557858.2016.1182126 27174778

[papt70063-bib-0104] Smith, J. , Flowers, P. , & Larkin, M. (2009). Interpretative phenomenological analysis. Theory, method and research. Sage Publications Ltd.

[papt70063-bib-0106] Smith, J. A. , Flowers, P. , & Larkin, M. (2022). Interpretative phenomenological analysis: Theory, method and research (2nd ed.). Sage.

[papt70063-bib-0107] Stänicke, L. I. (2021). The punished self, the unknown self, and the harmed self—Toward a more nuanced understanding of self‐harm among adolescent girls. Frontiers in Psychology, 12, 543303. 10.3389/fpsyg.2021.543303 PMC8064031 33897508

[papt70063-bib-0108] Stubbe, D. E. (2020). Practicing cultural competence and cultural humility in the care of diverse patients. Focus, 18(1), 49–51. 10.1176/appi.focus.20190041 PMC7011228 32047398

[papt70063-bib-0109] Sutton, J. (2007). Healing the hurt within: Understand self‐injury and self‐harm, and heal the emotional wounds. How To Books Ltd: Distributor Grantham Book Services Ltd.

[papt70063-bib-0111] Tabassum, R. , Macaskill, A. , & Ahmad, I. (2000). Attitudes towards mental health in an urban Pakistani community in the United Kingdom. International Journal of Social Psychiatry, 46(3), 170–181. 10.1177/002076400004600303 11075629

[papt70063-bib-0112] The British Psychological Society . (2017). Who psychologists work with. Practice Guidelines, 3.

[papt70063-bib-0114] Tofthagen, R. , Gabrielsson, S. , Fagerström, L. , Haugerud, L. , & Lindgren, B. (2021). Men who self‐harm—A scoping review of a complex phenomenon. Journal of Advanced Nursing, 78(5), 1187–1211. 10.1111/jan.15132 34931712

[papt70063-bib-0115] Tracy, S. J. (2010). Qualitative quality: Eight “big‐tent” criteria for excellent qualitative research. Qualitative Inquiry, 16(10), 837–851. 10.1177/1077800410383121

[papt70063-bib-0117] Vickers, M. H. (2000). Stigma, work, and “unseen” illness: A case and notes to enhance understanding. Illness, Crisis & Loss, 8(2), 131–151. 10.1177/105413730000800203

[papt70063-bib-0118] Wallace, S. , Nazroo, J. , & Bécares, L. (2016). Cumulative effect of racial discrimination on the mental health of ethnic minorities in the United Kingdom. American Journal of Public Health, 106(7), 1294–1300. 10.2105/ajph.2016.303121 PMC4984732 27077347

[papt70063-bib-0119] Warm, A. , Murray, C. , & Fox, J. (2003). Why do people self‐harm? Psychology, Health & Medicine, 8(1), 72–79. 10.1080/1354850021000059278 21888490

[papt70063-bib-0120] Watson‐Singleton, N. N. (2017). Strong black woman schema and psychological distress: The mediating role of perceived emotional support. Journal of Black Psychology, 43(8), 778–788. 10.1177/0095798417732414

[papt70063-bib-0121] Wijekoon, S. , & Peter, N. (2022). Examining racial, ethnic, and cultural diversity in occupational science research: Perspectives of persons of color. Journal of Occupational Science, 30(3), 322–341. 10.1080/14427591.2022.2119269

[papt70063-bib-0122] World Health Organization . (2021). Guidance on community mental health services: promoting person‐centred and rights‐based approaches. WHO. https://apps.who.int/iris/bitstream/handle/10665/341648/9789240025707‐eng.pdf?sequence=1&amp=&isAllowed=y

[papt70063-bib-0123] Worley, J. (2020). Self‐injury as an addictive disorder. Journal of Psychosocial Nursing and Mental Health Services, 58(6), 13–16. 10.3928/02793695-20200513-03 32463908

